# Artificial molecular machines: Design and observation

**DOI:** 10.1002/smo.20230015

**Published:** 2023-10-23

**Authors:** Shu Zhang, Yi An, Xu‐man Chen, Quan Li

**Affiliations:** ^1^ Institute of Advanced Materials and School of Chemistry and Chemical Engineering Southeast University Nanjing Jiangsu Province China

**Keywords:** artificial molecular machines, mechanically interlocked molecules, molecular motors, molecular switches, supramolecular chemistry

## Abstract

Natural molecular machines have inspired the development of artificial molecular machines, which have the potential to revolutionize several areas of technology. Artificial molecular machines commonly employ molecular switches, molecular motors, and molecular shuttles as fundamental building blocks. The observation of artificial molecular machines constructed by these building blocks can be highly challenging due to their small sizes and intricate behaviors. The use of modern instrumentation and advanced observational techniques plays a crucial role in the observation and characterization of molecular machines. Furthermore, a well‐designed molecular structure is also a critical factor in making molecular machines more observable. This review summarizes the common methods from diverse perspectives used to observe molecular machines and emphasizes the significance of comprehending their behaviors in the design of superior artificial molecular machines.

## Graphical abstract

Artificial molecular machines have undoubtedly been an area essential in the past half century. The comprehension and investigation of molecular machines' motion are inexorably linked to modern instrumentation, advanced observational techniques and well‐designed molecular structures. On the other hand, such observations also inspire improved designs of artificial molecular machines. Here, we summarize common methods and strategies from different perspectives for observing molecular machines.

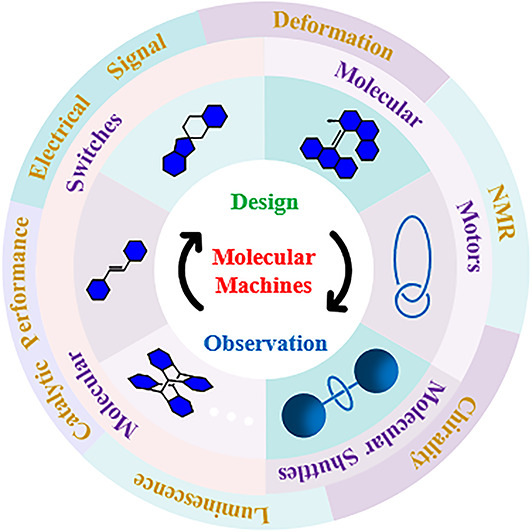

## 1 INTRODUCTION

**FIGURE 1:**
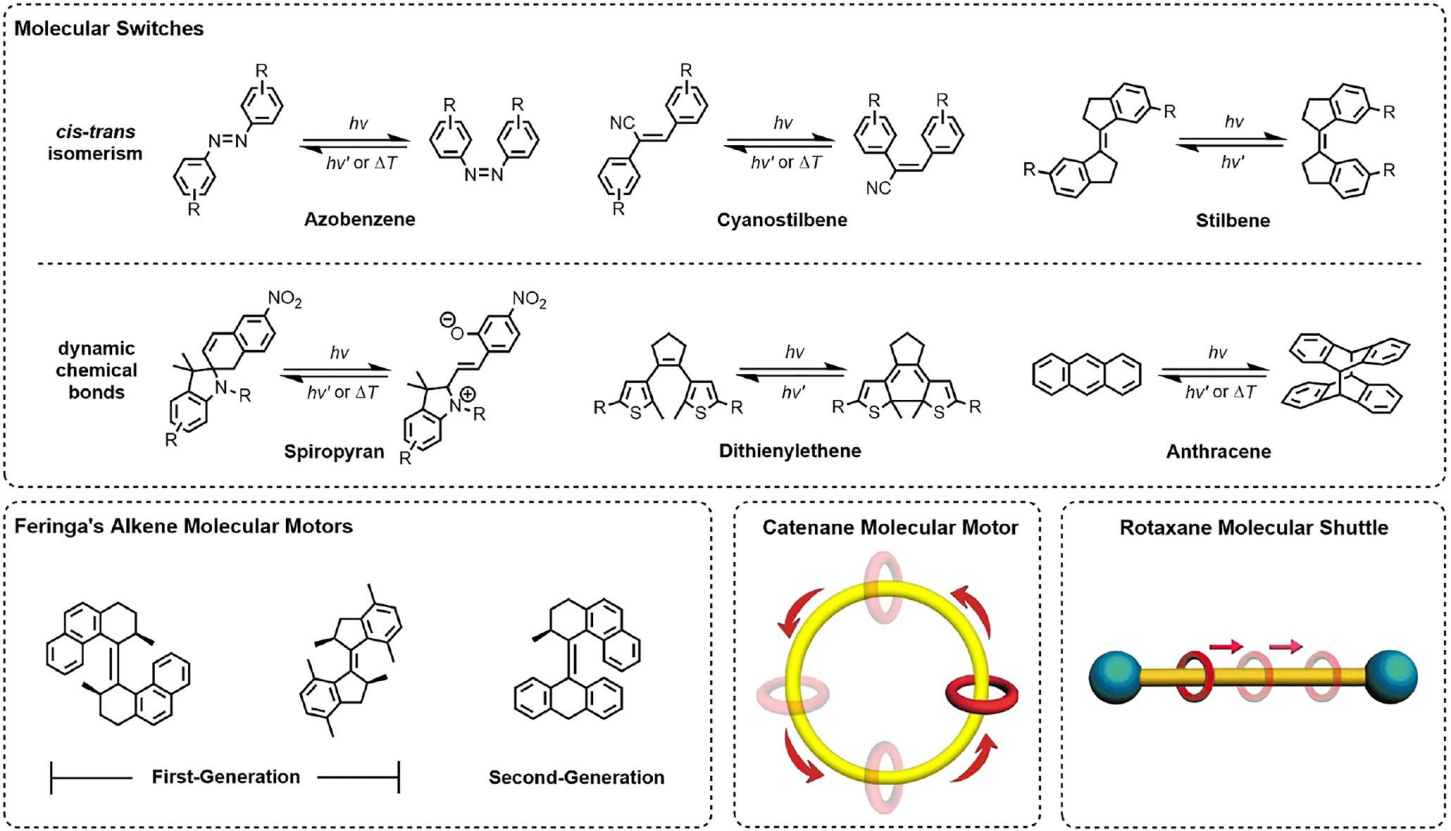
Common basic molecular machine units.

Molecular machines are ubiquitous in nature and perform a wide range of functions in living organisms.[^1^, ^2^] These machines are composed of biological molecules[^3^, ^4^, ^5^] and are capable of performing specific tasks with remarkable precision and efficiency. Examples of molecular machines in nature include enzymes that catalyze chemical reactions,[^6^, ^7^, ^8^, ^9^] motor proteins that transport cargo within cells,[^10^, ^11^, ^12^] and ribosomes that synthesize proteins.[^13^, ^14^] The study of these natural molecular machines has not only provided insights into the inner workings of living organisms but has also inspired the development of artificial molecular machines,[^15^, ^16^] which is the subject of the 2016 Nobel Prize in Chemistry.[^17^, ^18^] The field of artificial molecular machines has emerged as a promising area of research that spans across chemistry, physics, and materials science. These tiny machines are constructed from individual molecules, and they have the potential to revolutionize several areas of technology, including the synthesis of complex molecules,[^19^, ^20^] drug delivery,^[21]^ artificial muscles,[^22^, ^23^] and energy conversion.[^24^, ^25^, ^26^] Although there are a number of complex artificial molecular machines have been designed, their constructions are mainly based on the basic molecular machine units, which typically include molecular switches, molecular motors and molecular shuttles (Figure 1).

Molecular switches[^27^, ^28^, ^29^] have two or more stable states and can change their state in response to external stimuli, such as light, pH, or temperature. At present, molecular switches can be divided into two main categories: Some molecular switches utilize *cis*‐*trans* isomerism of the double bond,^[30]^ such as azobenzene,[^31^, ^32^, ^33^, ^34^] cyanostilbene,[^35^, ^36^] and stilbene; others utilize dynamic chemical bonds that can break and reform under certain conditions, such as spiropyran,[^37^, ^38^] ditheienylethene[^39^, ^40^, ^41^, ^42^] and anthracene.^[43]^ Among molecular motors, alkene molecular motors are relatively common. Compared to molecular switches, although both use the *cis*‐*trans* isomerism, alkene molecular motors can rotate unidirectionally around the alkene,^[44]^ due to the well‐designed large steric hindrance and chiral groups. The second generation of alkene molecular motors has recently been found to have better photostability than the first generation,^[45]^ opening up wider application prospects. Another type of molecular motor is based on a catenane structure,^[46]^ in which two rings are mechanically interlocked, with one ring operating in a unidirectional rotation along a larger ring. Finally, molecular shuttles^[47]^ usually refer to the fact that a ring in a rotaxane can move unidirectionally from one location to another along the axle. The rotaxane structure can restrict the freedom of movement of the components in certain directions, while allowing large‐amplitude displacement in other directions.^[48]^

Whether it is a molecular machine in nature or an artificial molecular machine, the main concern is whether and how it moves. In the case of molecular machines in nature, especially in our bodies, failure or malfunction can lead to various diseases.^[49]^ Similarly, observing the movement of artificial molecular machines is of great importance in the field of nanotechnology. By studying the behavior of these molecular machines, researchers can also gain insights into how they work and how they can be optimized for specific applications. In addition, understanding how they operate can help us better design artificial molecular machines for more applications.^[50]^ Overall, observing artificial molecular machines is crucial to advance our understanding of nanotechnology and unlocking their potential to solve real‐world problems.

However, observing artificial molecular machines can be extremely challenging due to their small size and complex behavior.^[51]^ These machines are often on the scale of just a few nanometers, making them difficult to observe using traditional microscopy techniques. Furthermore, their behavior is highly dependent on their environment and can be affected by factors such as temperature, pressure, and the presence of other molecules. This means that careful control of experimental conditions is necessary to ensure accurate observations. In addition, many artificial molecular machines are highly dynamic and can change shape rapidly, further complicating efforts to observe and characterize them. Despite these challenges, advances in instrumentation and imaging techniques^[52]^ combined with well‐designed molecular structures are enabling scientists to make progress in observing these machines and understanding their behaviors. In this review, we summarize common methods and strategies from different perspectives for observing molecular machines, hoping to inspire scientists to better design artificial molecular machines.

## 2 DEFORMATION

Deformation is direct evidence that a molecular machine is working. The deformation of molecular machines can be shown as microscopic and macroscopic deformation. Microscopic deformation cannot be seen with the naked eye, so advanced instruments are usually used, whereas macroscopic deformation can be seen with the naked eye, such as stretching and shrinking of materials. In addition, the amplification of microscopic deformation can also induce macroscopic deformation in specific materials.

### 2.1 Microscopic deformation

**FIGURE 2:**
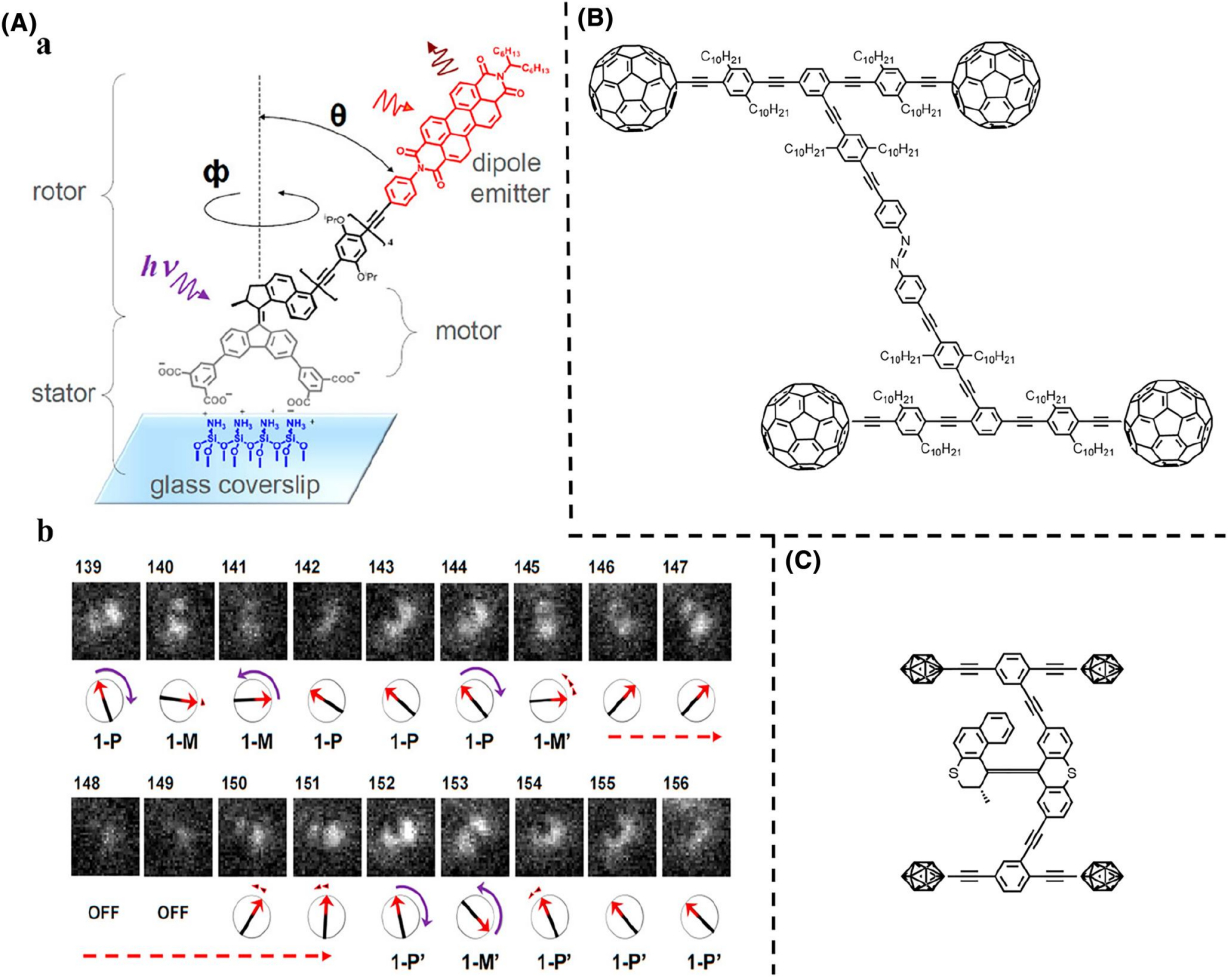
(A) (a) Schematic illustration and chemical structure of the surface‐bound alkene molecular motor. (b) Experimental defocused patterns of surface‐bound motors and the relative nanomechancial state in each frame by calculation. *Copyright © 2017, American Chemical Society.* (B) Chemical structure of the molecular car based on the azobenzene molecular switch. (C) Chemical structure of the molecular car based on the alkene molecular motor.

Microscopy is one of the most important tools for observing the microscopic world. Optical microscope (OM) tends to be more tolerant of complex measurement environments due to its high temporal resolution, but low spatial resolution due to diffraction limitations. Thus, large molecular machines such as kinesin and dynein, or fluorescently labeled biomolecules, are commonly observed by OM.[^53^, ^54^, ^55^] For smaller molecular machines, OM can be improved by pulling the molecules away from the focal plane, called defocused OM.^[56]^ The patterns formed beyond the focal plane can be correlated with the orientation of the dipole of a single molecule. For instance, Hofkens et al.^[57]^ modified an overcrowded alkene molecular motor and bound it to a glass surface (Figure 2Aa). The rotation of the individual alkene molecular motor can be controlled by alternating photoisomerization and thermal relaxation steps. Defocused OM was used to record the changes in dipole direction of the alkene molecular motor with integration time of ∼200 ms. The photoisomerization steps and the thermal relaxation steps can be distinguished by different changes in the dipole angle (Figure 2Ab).

Notwithstanding, OM still has too many limitations in the observation of molecular machines. The spatial resolution is far from the atomic scale, so changes in molecular structure cannot be directly observed, and the distinct microscopic deformation is still difficult to determine. The scanning tunneling microscope (STM), an instrument that uses the tunneling effect of quantum theory to detect the surface structure of matter, has become an important measurement and processing tool in nanotechnology.[^58^, ^59^, ^60^] Due to its higher resolution, STM allows clearer observation of molecular machines during their microscopic deformation, such as molecular cars. Tour et al.^[61]^ first reported a nanocar, with four ‘wheels’–C_60_, that could be seen moving along the axis of the gold surface by molecular thermodynamic motion under an STM. The same group then installed an azobenzene molecular switch on the nanocar to provide power, making it a light‐driven nanocar (Figure 2B).^[62]^ The incorporation of the photo‐responsive azobenzene chassis allowed for potential wormlike motion to accompany the rolling behavior of the wheels. Although, they later designed a nanocar based on an alkene molecular motor, the motor will have sufficient energy to rotate and thus propel the nanocar on a surface yet to be determined (Figure 2C).^[63]^

**FIGURE 3:**
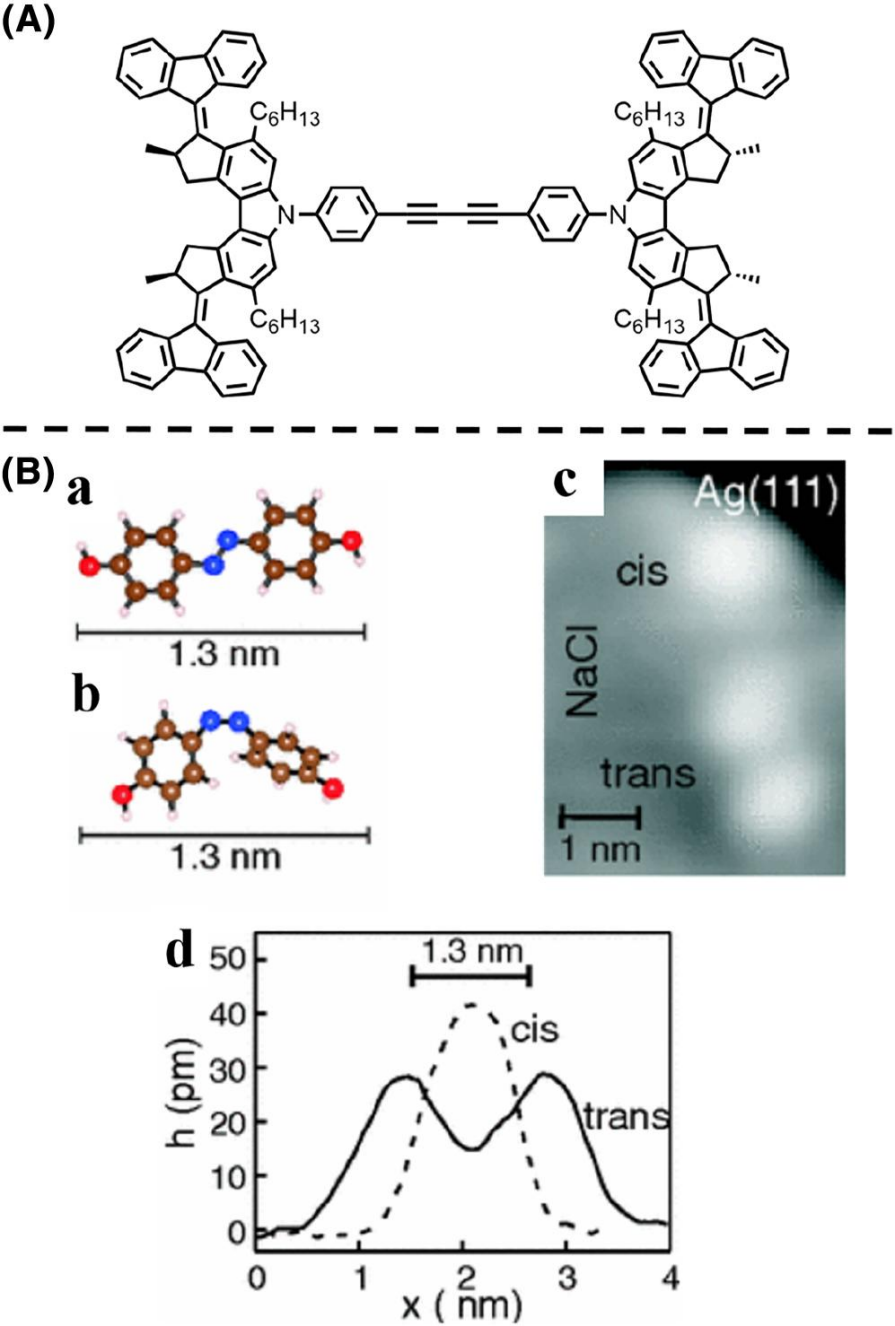
(A) Chemical structure of the molecular car. (B) Ball‐and‐stick models of (a) the *trans*‐ and (b) *cis*‐isomer of the azobenzene molecular swingers as optimized in gas phase. (c) STM image of the *trans*‐ and *cis*‐isomer on a NaCl double layer on Ag(111). (d) The apparent heights of the *trans*‐ and *cis*‐isomer. *Copyright © 2012, American Physical Society.*

Subsequently, Feringa et al.^[64]^ demonstrated a different strategy in which crowded alkene molecular motors acted as molecular wheels rather than the body of the car (Figure 3A). These motors, which can be triggered by a tunneling current from the STM, allowed the car to move in a specific direction. By adjusting the chirality of the individual motor units, the molecular car can move in a linear or random trajectory, or remain stationary due to internal compensation. A positive voltage was also applied to the car to demonstrate an electrically driven motion. However, the molecular car cannot be actuated due to high energy barriers caused by the intervention of the substrate. To overcome this issue, two of the wheels were cut off to reduce the energy barriers, thus enabling the actuation of the molecular car.

In addition to molecular cars, molecular swingers are molecules that can achieve molecular‐scale swinging through conformation/configuration changes under external stimuli such as electrochemical reactions and light excitation. By attaching different number of tert‐butyl ligands (legs) to the azobenzene molecular switch, Morgenstern et al.^[65]^ reported that the photomechanical activity of a molecular swinger was enhanced by STM characterization (Figure 3Ba,b). The tert‐butyl legs can lift the molecules away from the surface, thereby reducing the molecule‐surface coupling. Azobenzene molecular swingers were deposited on the NaCl layer grown on a clean Ag(111) surface at 17 K, clearly presented under STM (Figure 3Bc). The isomerization from *trans*‐to *cis*‐isomer of almost all molecules on the surface was triggered after illumination with 365 nm light for 18 h, along with the change of the apparent heights (Figure 3Bd).

### 2.2 Macroscopic deformation

**FIGURE 4:**
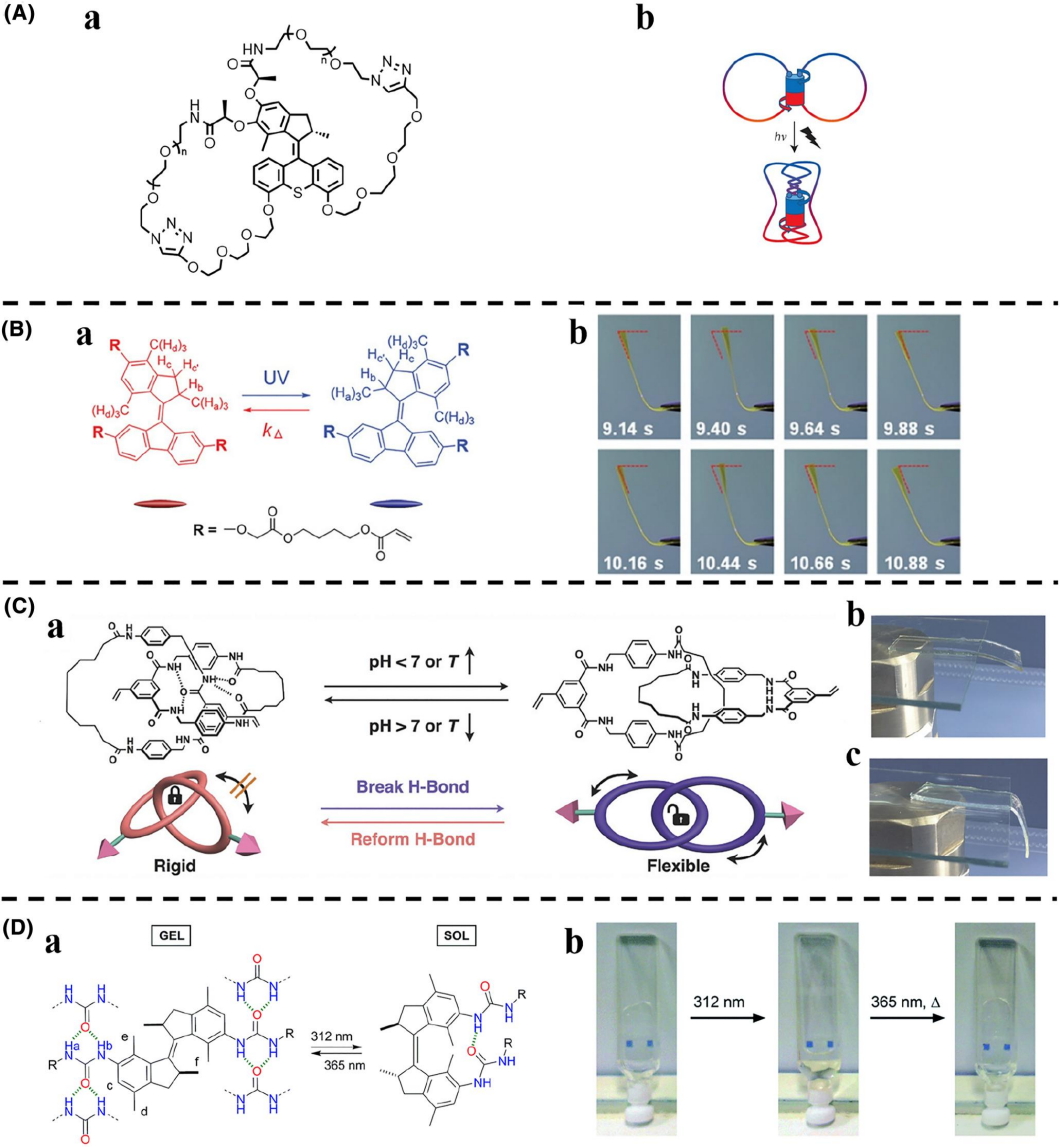
(A) (a) Chemical structure of the cross‐linked polymer‐motor conjugates. (b) Schematic representation of the contraction of the cross‐linked polymer‐motor conjugates under UV irradiation. (B) (a) Chemical structure of the motor‐linked liquid crystalline network monomer in stable form and unstable form. (b) Time‐resolved deflection angle of the liquid crystalline network. *Copyright © 2021 Wiley‐VCH GmbH*. (C) (a) Structural change of the catenane‐linked gel monomer under external pH and temperature stimuli. Photographs were the gel (b) before and (c) after swelling in acidic solution. *Copyright © 2017 WILEY‐VCH Verlag GmbH & Co. KGaA, Weinheim*. (D) (a) Photoisomerization behavior and predicted urea hydrogen bonding pattern of the *trans* and *cis* bis‐urea alkene molecular motor. (b) Photographs of the *trans‐*motor before and after 15 min irradiation with 312 nm light, showing gel dissolution. The resulting solution was irradiated for 15 min with 365 nm light and gently heated to reform the gel upon slow cooling. *Copyright © 2016, Royal Society of Chemistry*.

Artificial molecular machines are designed to mimic the machines in real life that can perform macroscopic motions to deliver energy to the environment, such as motors and pumps. By chemically cross linking with polymers, tiny molecular machines can produce visible macroscopic deformation as well. Giuseppone et al.^[66]^ synthesized cross‐linked polymer‐motor conjugates in the form of a gel in toluene at 10% wt/wt (Figure 4Aa). The study revealed that the unidirectional rotations of the alkene molecular motors, driven by simple irradiation with ultraviolet (UV) light and working out of equilibrium, were able to produce a strong macroscopic contraction at room temperature (Figure 4Ab). This was further confirmed at the microscopic scale through atomic force microscopy (AFM), which is an analytical instrument that can be used to study the surface structure of solid materials.[^67^, ^68^] Images of the gel surface showed a significant change in the average size of pore diameters when comparing the initial state with the contracted state. These observations indicated that the macroscopic contraction resulted from the twisting of the entangled polymer chains in the strained gel, leading to more empty spaces between densely wound regions.

Vibration is another common form of macroscopic deformation. Yang et al.^[69]^ developed a novel liquid crystalline network linked with alkene molecular motors that can still undergo photoinduced *cis*‐*trans* isomerization after crosslinking (Figure 4Ba). The dynamic change of the molecular motor controlled the mechanical properties of the liquid crystalline network without altering the order of the liquid crystalline matrix. As a result, the macroscopic vibrational behavior can be triggered by UV light (Figure 4Bb). Furthermore, when the molecular motor was doped in a liquid crystalline network with one side oriented parallel and the other side oriented vertically, the liquid‐crystalline network can vibrate with different amplitudes under varying intensities of UV light. The doping concentration of the molecular motor can also effectively control the vibrational behavior of the liquid crystalline network.

Form change of materials can also be considered as macroscopic deformation, such as hard material versus soft material, gel form versus solution form, etc. Huang et al.^[70]^ demonstrated a gel prepared by the thiol‐ene “click” reaction between bisvinyl[2]catenane and thiol‐derivatized poly(ethylene glycol) (Figure 4Ca). The catenane crosslinker was responsive to external pH or temperature stimuli due to the presence of hydrogen bonding. The strong hydrogen bonding restricted the rotation and movement of the crosslinker, giving it a rigid property (Figure 4Cb). However, when the hydrogen bonding was broken, the crosslinker became flexible. As a result, the gel can be reversibly switched between tough and soft states under stimuli (Figure 4Cc).

Also using hydrogen bonding, Feringa et al.^[71]^ presented a low molecular weight urea‐containing gel derived from an overcrowded alkene molecular motor (Figure 4Da). For the *trans* isomer of this gel, intermolecular urea hydrogen bonding existed, whereas the *cis* isomer indicated the possible formation of intramolecular hydrogen bond. Thus, irradiation of the gel triggered *trans*‐to‐*cis* isomerization and consequently a gel‐sol phase transition (Figure 4Db). This process can be completely reversed by changing the irradiation wavelength.

### 2.3 Microscopic deformation driving macroscopic deformation

Liquid crystal[^72^, ^73^, ^74^] is a type of molecule that exhibits a liquid crystalline state under certain conditions. Typically, a liquid crystal molecule has an anisotropic shape, such as a rod or a disc. When heated to a certain temperature or dissolved in a solution of a certain concentration, a liquid crystal presents a special state that combines the fluidity of the liquid and the anisotropy of the crystal. According to its properties, liquid crystal is an effective way to amplify the invisible deformation of molecular machine.^[75]^

In 2006, Feringa et al.^[76]^ discovered a novel light‐responsive deformation of an alkene molecular motor doped liquid crystal material. They introduced the alkene molecular motor into a nematic liquid crystal at a low weight ratio of 1%. By coating the cholesteric liquid crystal mixture on the surface, periodic liquid crystal stripes were formed. Upon exposure to UV light, the stripes of the cholesteric liquid crystal coating underwent a unidirectional rotation due to the photoisomerization of the molecular motor. It was observed that the rotated stripes could drive the rotation of a micro‐sized rod, indicating that the nanoscale motor could actuate a microscale molecular machine through the amplification effect of the liquid crystal matrix.

**FIGURE 5:**
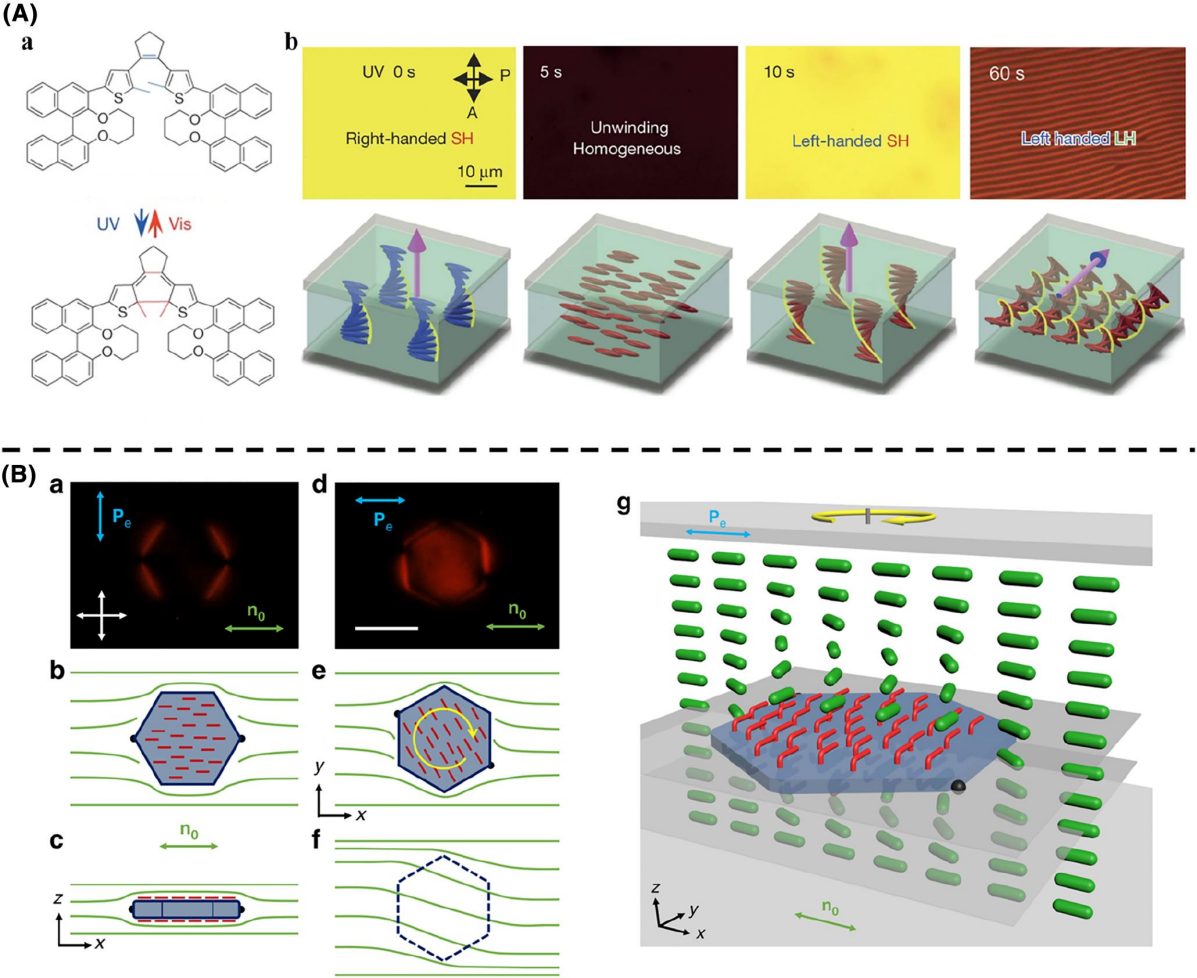
(A) (a) Chemical structure of the dithienylcyclopentene molecular switch in open form and closed form. (b) UV irradiation of the switch in a planar cell transformed the helical superstructure from its original standing helices, through the unwound homogeneous state and the standing helix arrangement with opposite handedness, to the lying helix arrangement. *Copyright © 2016, Springer Nature Limited.* (B) The self‐assembled colloidal azobenzene molecular switches in a nematic liquid crystal. (a) POM image obtained under red light when the platelet was illuminated by linearly polarized blue light. (b, c) Corresponding schematics of *trans*‐state azobenzene moieties orientations (red rods) within the self‐assembled monolayers in the plane parallel to cell substrates and in the cell's vertical cross‐section. (d) POM image of the same particle as in a, but when it rotated under blue‐light illumination. (e, f) Corresponding schematics of azobenzene orientations in the plane of platelets and in the plane beneath. (g) Three‐dimensional schematic of the molecular‐colloidal light‐driven switches between two confining glass plates, with the green rods represent liquid crystal molecule and the azobenzene switches of self‐assembled monolayers shown in red.

Similarly, Li et al.^[77]^ doped a dithienylcyclopentene molecular switch (1.2 mol%) into a cholesteric liquid crystal system prepared from the achiral nematic liquid crystal E7 (Figure 5Aa). Under polarized optical microscopy (POM), which can test the anisotropy and birefringence of the sample, it was initially in a bright state, due to the expected Grandjean planar texture‐standing helices. After UV light irradiation for 5 s, the bright state changed to a dark state, corresponding to the unrolled nematic phase resulting from the homogeneous alignment of the liquid crystal molecules (Figure 5Ab). After 10 s of irradiation, the bright state reappeared, accompanied by simultaneous in‐plane rotation of the stripes and pitch contraction until the system reached the photostationary state. Thus, the helical axis direction of the cholesteric liquid crystal can be controlled in three dimensions by light alone, enabling reversible, large‐area, two‐dimensional in‐plane beam steering.

Smalyukh et al.^[78]^ used a self‐assembled colloidal azobenzene molecular switch, which exhibited repetitive light‐driven rotation of transparent microparticles immersed in a liquid crystal and driven by continuous exposure to unstructured ∼1 nW light (Figure 5Ba,b). The optical axis of the liquid crystal was mechanically coupled to the surface of the particle, and they rotated together as the polarization of the light changed. The rotating particle twisted the arrangement of the liquid crystal matrix, which in turn changed the polarisation of the traversing light (Figure 5Bb‐g). This feedback mechanism enabled a continuous opto‐mechanical cycle, resulting in a unidirectional particle rotation whose handedness and frequency were robustly controlled by the polarisation and intensity of the light.

## 3 Nuclear Magnetic Resonance

**FIGURE 6:**
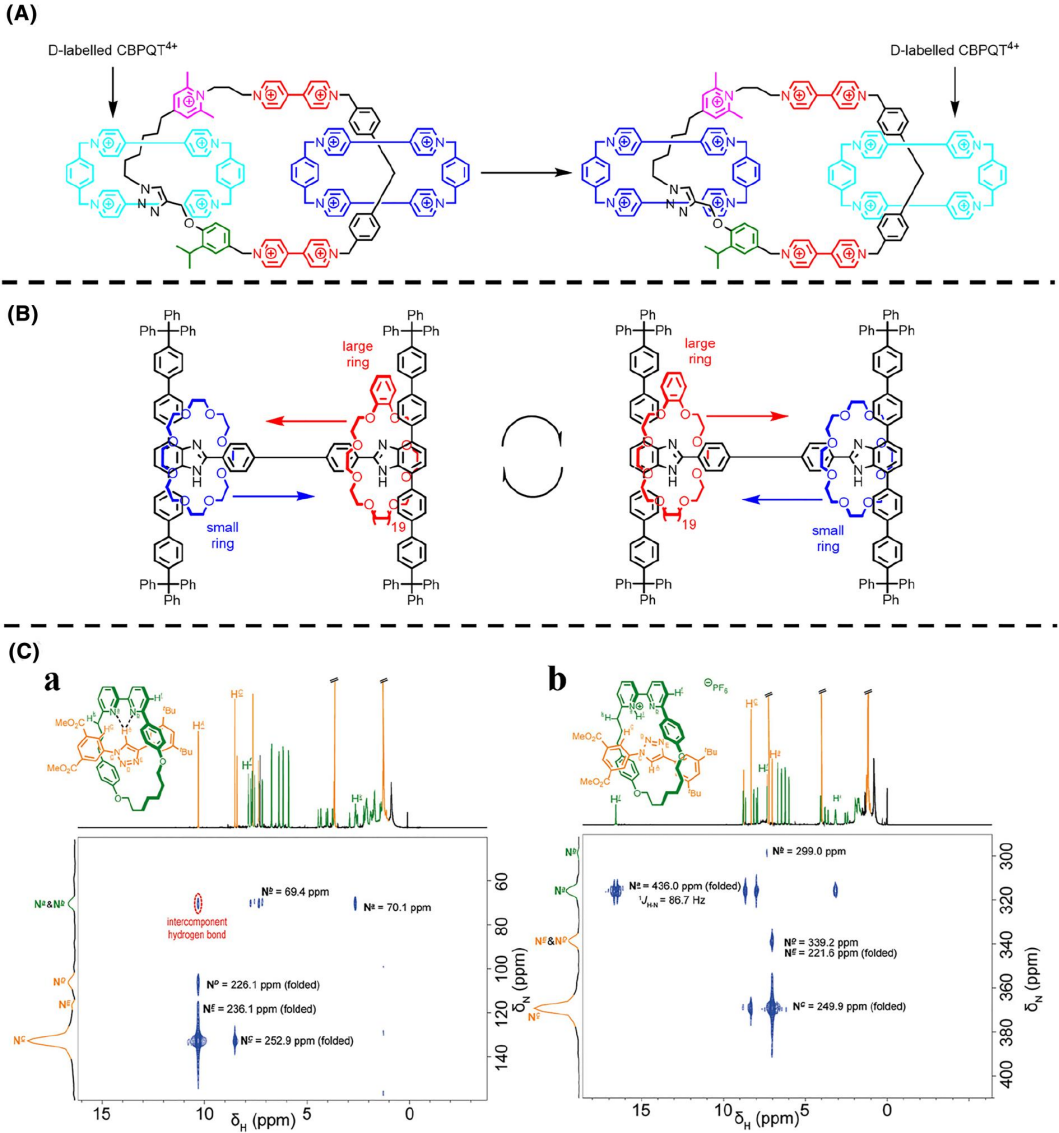
(A) Chemical structure of the electric catenane molecular motor with a D‐labeled CBPQT^4+^ macrocycle. (B) Chemical structure of the ring‐through‐ring molecular shuttle. (C) ^1^H‐^15^N HMQC spectra (500–51 MHz, CDCl_3_, 263 K, 50 mM) of the rotaxanes. *Copyright © 2023, American Chemical Society.*

In fact, most molecular machines have not been able to produce visible deformation. Therefore, more advanced instruments are needed to characterize structural changes at the molecular level. Nuclear magnetic resonance (NMR) refers to the phenomenon whereby an atomic nucleus with a fixed magnetic moment absorbs or releases energy in the form of electromagnetic waves under the action of a constant magnetic field and an alternating magnetic field, resulting in a transition of the atomic nucleus, while generating an NMR signal. Today, NMR technology is widely used to study the chemical environment of the atoms of various molecules, including molecular machines. From the earliest molecular machines, such as Stoddart and Suavage's rotaxane shuttle,[^79^, ^80^] to the present day, NMR has always played an important role in investigating the motion of molecular machines. In general, the state and conservation of molecular machines can be determined by simple ^1^H NMR. Furthermore, to study complex molecular machines, deuterium substitution is a powerful tool, due to the disappearance of deuterium in ^1^H NMR. Stoddart et al.^[81]^ designed an electric molecular motor based on^[3]^ catenane, consisting of two smaller cyclobis(paraquat‐p‐phenylene) (CBPQT^4^^+^) rings^[82]^ strung on a 50‐membered ring (Figure 6A). On the 50‐membered ring, two viologen (V^2+^) units were separated by a bis(4‐methylenephenyl) methane unit, which can serve as recognition sites for the two reduced ring CBPQT^2(+•)^ when reduced to a free radical cation state (V^+•^). The rest of the 50‐membered ring consisted of a chain containing a total of 11 methylene and 1 oxygen atom, as well as an isopropylphenylene spatial barrier, a triazole ring, and a 2,6‐dimethylpyridinium coulomb barrier. Due to the redox properties of the V^2+/+•^ unit and the two CBPQT^4+/2(+•)^ rings in the 50‐membered ring, the oxidized [3]catenane motor can be converted to the reduced state, while the two CBPQT^2(+•)^ rings also moved, thereby being recognized by the V^+•^ unit through free radical pairing interactions to undergo a unidirectional rotational motion along the larger 50‐membered ring. To confirm the unidirectionality of the rotation of the electric molecular motor, deuterium labeling was carried out by introducing deuterium atoms into one of the two CBPQT^4+^ rings, thus distinguishing the two CBPQT^4+^ rings. The corresponding ^1^H NMR spectrum proved that the two CBPQT^4+^ rings had indeed completed a 180° one‐way rotation after undergoing a redox cycle, and the completion rate was also calculated to be approximately 85%.

By taking advantage of temperature to control the rate of motion, variable temperature NMR can also be effectively used to observe molecular machines. Loeb et al.^[83]^ synthesized a novel molecular shuttle, which mainly contained a rigid H‐shaped axle, two benzimidazole recognition sites, and two polyether macrocycles: one was a smaller, 24‐crown 8‐ether (24C8); the other was a larger, benzo‐[n]‐crown 8‐ether, where *n* was 30 (B30C8) or 42 (B42C8) (Figure 6B). It was found that the smaller rings in this molecular shuttle can pass through the larger rings to achieve a rare “ring through the ring” molecular shuttle motion. In order to further investigate this molecular shuttle phenomenon, variable temperature ^1^H NMR experiments were conducted on the [3]rotaxane in different solvents (toluene *d*_8_ and DMSO *d*_6_). At low temperatures, the rate of translational motion was very slow, resulting in the clear identification of the peaks of the two occupied recognition sites. As the temperature increased, the two peaks gradually became broad, and eventually became distinct single peaks above room temperature. This indicates that at higher temperature the large and small rings on the axis can rapidly shuttle at the two benzimidazole recognition sites, faster than the detection time of ^1^H NMR. The only reasonable explanation for this situation is that the small ring passes through the large ring and undergoes a “ring through the ring” molecular shuttle motion.

2D NMR is a testing technique that extends from conventional 1D NMR, greatly reducing the degree of crowding and overlapping of spectral lines, and providing new information on the interrelationship between nuclear spins, which is particularly useful for the analysis of complex macromolecules. In supramolecular chemistry, 2D NMR, such as NOESY/ROSEY, provides direct evidence related to the position of different components.[^84^, ^85^, ^86^] With the rapid development of 2D NMR, several nitrogen atoms coupling 2D NMR (IMPACT‐HMNBC, JNNH_NN_‐COSY, SOFAST‐HMBC, SOFAST‐HMQC, etc.) have been employed to study biomolecules' folding and assembly, especially for ^15^N isotope‐enriched molecules, like proteins.[^87^, ^88^] However, these techniques are less widely used in the study of molecular machines and are expected to provide alternative information about molecular machines. Wilson et al.^[89]^ demonstrated that *J* coupling between hydrogen‐bonded nitrogen and hydrogen nuclei in ^1^H‐^15^N HMQC experiments allowed the observation of intra and intermolecular hydrogen bonding in rotaxanes. In the ^1^H‐^15^N HMQC spectrum of [2]rotaxane, a downfield shift for the triazole proton from 8.24 to 10.30 ppm was observed for the rotaxane relative to the component axle, due to hydrogen bonding (Figure 6Ca). Correlation was observed between the triazole proton and the bipyridine nitrogen atoms, indicating a through‐space interaction. This through‐space correlation signified direct orbital communication between the hydrogen and nitrogen atoms, that is, a proximity‐enforced hydrogen bond. The ^1^H‐^15^N HMQC experiment can also be used to identify stimulation‐induced structural changes in the [2]rotaxane. Upon the protonation of the [2]rotaxane to form the [2]rotaxane [HPF_6_] salt, a co‐conformational change was induced, which resulted in the resonance for the triazole proton shifting upfield to 7.02 ppm as it no longer participated in hydrogen bonding, while the bipyridine nitrogen atoms shifted downfield relative to the unprotonated macrocycle, indicating protonation (Figure 6Cb). Although ^1^H‐^15^N HMQC has not yet been used in a real molecular machine system, this example fully demonstrated its potential in the study of hydrogen bonding related molecular machines.

## 4 CHIRALITY

Chiral molecules play a crucial role in organisms, and many chemical reactions that drive cells only work with molecules that have specific chiral configurations. As one of the central themes of chemistry, chirality is also widespread in molecular machines. Changes in chirality caused by the motion of molecular machines will lead to many phenomena, such as dynamic self‐organized helical superstructures (i.e., cholesteric liquid crystals),[^90^, ^91^, ^92^, ^93^, ^94^, ^95^] tunable circularly polarized luminescence (CPL),^[96]^ and controllable catalytic performance. These induced phenomena are discussed in the corresponding chapters. Here, we focus only on the chirality transformation of molecular machines.

**FIGURE 7:**
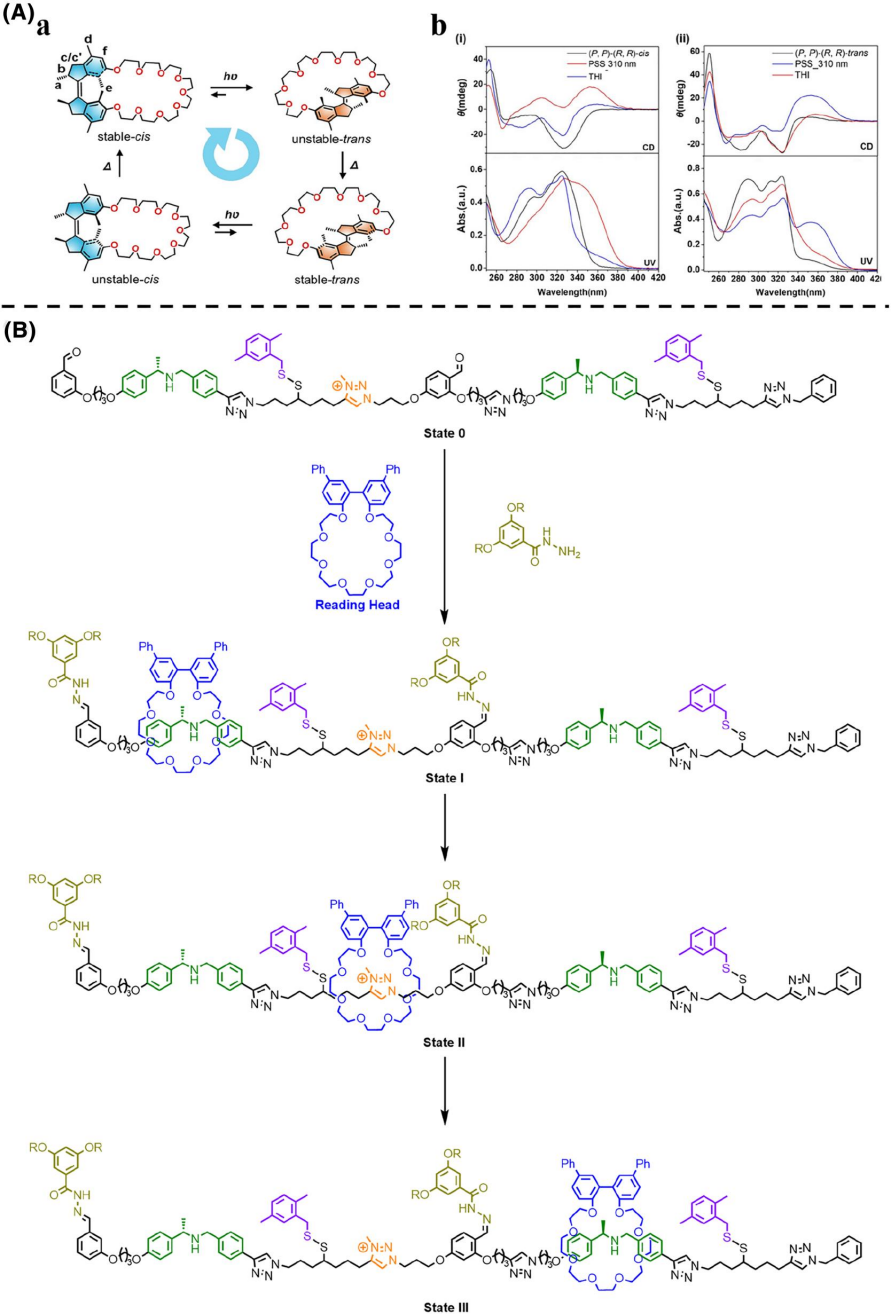
(A) (a) The four‐step rotation in an intramolecular confined space of the alkene‐motorized macrocycle. (b) (i) The CD (upper) and UV/Vis absorption (lower) spectra of the (*P*, *P*)‐(*R*, *R*)‐*cis* state before and after photoisomerization. (ii) The CD and UV/Vis absorption spectra of (*P*, *P*)‐(*R*, *R*)‐*trans* state before and after photoisomerization. (B) Chemical structure of the rotaxane shuttle‐based molecular tape and the pulse‐fueled transport of the macrocycle onto, along and off the molecular tape from state 0, state I, state II and state III.

When the first unidirectional alkene molecular motor was reported by Feringa,^[97]^ the chirality of the molecular motor changed with the rotation. Using circular dichroism (CD), which can measure the difference between the absorption of left and right circularly polarized light, the signal corresponded to each rotated state, clearly demonstrating the motion of the molecular motor. Among the four rotational states, the (*M*, *M*)‐*cis* isomer had the opposite CD signal to the others, indicating that its stereochemical environment had a significant difference, which can be used in dynamic stereoselective host‐guest systems. Similarly, Tian et al.^[98]^ presented an alkene molecular motor derived crown ether macrocycle (Figure 7Aa). The unidirectional rotation of the macrocycle also involved a four‐step rotation cycle consisting of two photostationary states (PSS) and two thermal helix inversion (THI) steps. It was found that the chirality reversal of the macrocycle occurred during the rotation (Figure 7Ab). When a (*R*)‐enantiomer of the guest was added, the (*M*, *M*)‐*cis* state of the macrocycle was favored to be bound while the (*S*)‐enantiomer was more strongly bound to the (*P*, *P*)‐*cis* state. Due to the dynamic isomerization of the (*P*, *P*)‐*cis* macrocycle to (*M*, *M*)‐*cis* upon photochemical and thermal steps, this macrocycle can modulate its binding affinity and stereoselectivity for different enantiomers of the chiral guest.

Chiral signals can also be used to store information. Leigh et al.^[99]^ demonstrated a novel tape‐like molecular ratchet. The rotaxane ratchet consisted of a crown ether–the reading head–threaded onto a molecular strand–the memory tape–with three compartments separated by a removable barrier (Figure 7B). These barriers could be opened by a pH pulse, in the form of a strong acid, which quickly decomposed and closed the barriers again. The ratchet was pushed forward and prevented from moving backward due to its higher binding affinity to the next compartment site. The first and last compartments contained chiral binding sites, a mirror image of the other, while the middle one was achiral. Although the crown ether was achiral, it can adopt a chiral arrangement when it interacted with a chiral binding site. The state of the molecular machine could be read from the CD response in the chromophore built into the structure of the crown ether. Three different signals can be observed: When the crown ether bound to the achiral *N*‐methyltriazolium unit in other parts of the tape, this asymmetry was not induced and there was no CD response. The signals were identified as +1 and −1 when the crown ether bound to the (*R*)‐ and (*S*)‐enantiomers of the *N*‐benzyl‐α‐methylbenzylammonium sites, respectively. Therefore, this system allowed the non‐destructive reading out of the sequence of stereochemical information “programmed” into the molecular tape by obtaining the information as a string of digits based on the observed CD responses.

## 5 LUMINESCENCE

Molecular machines are able to change their own light, or luminescence when they are set in motion, due to the coupling of mechanical motion and electronic properties of the molecules.^[100]^ Some molecular machines are designed to switch between different states, which can be accompanied by changes in luminescence, allowing changes in the state of molecular machines to be measured and monitored.[^101^, ^102^] Important changes in luminescence include wavelength and CPL.

### 5.1 Wavelength

The motions of some basic molecular machine units with emissions undergo wavelength changes as they move. For example, the non‐fluorescent spiropyran molecular switch can undergo photoisomerization to the fluorescent ring‐opening merocyanine. The merocyanine can then revert to the non‐fluorescent spiropyran when exposed to visible light.[^103^, ^104^]

**FIGURE 8:**
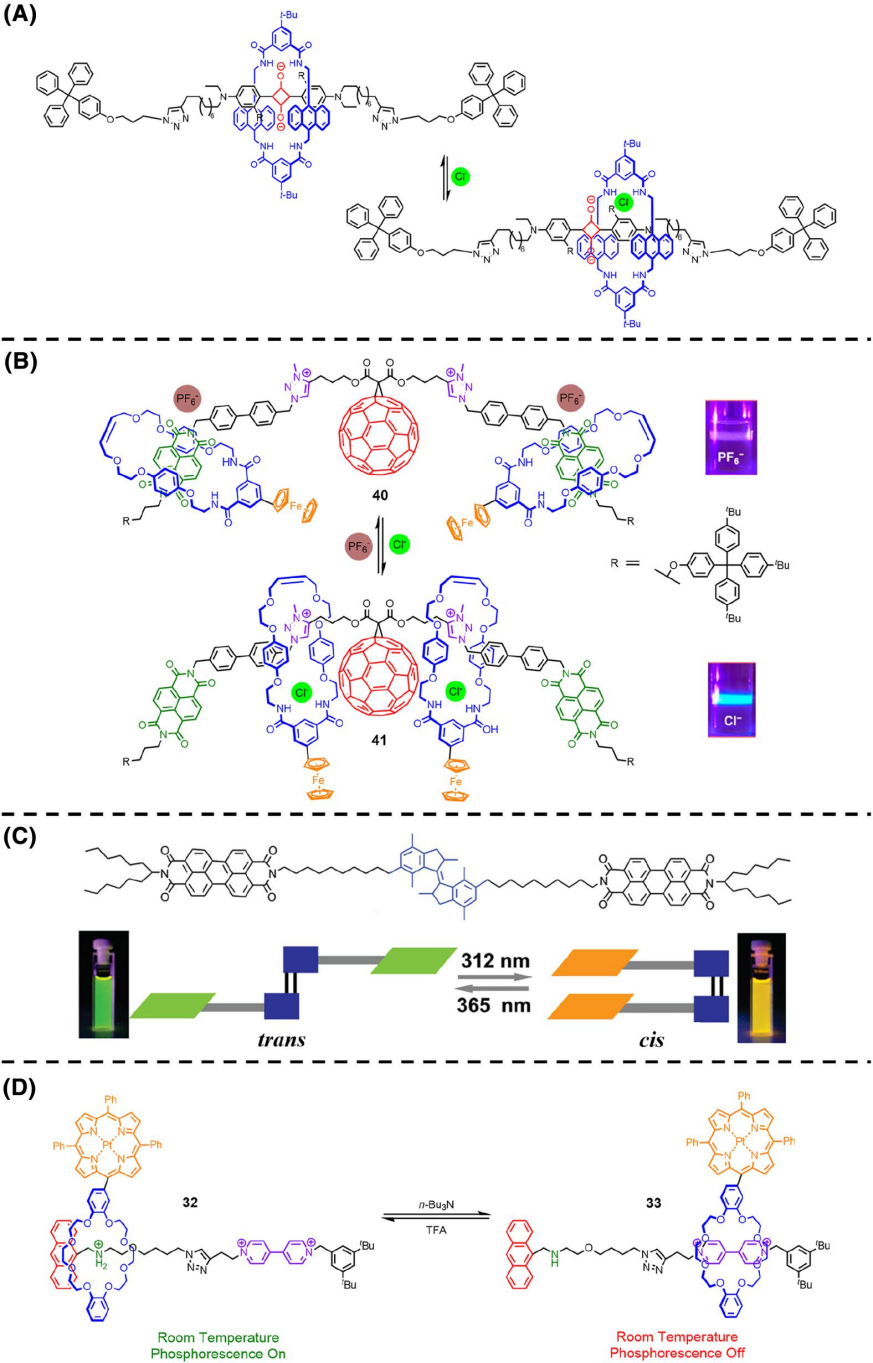
(A) Chemical structure of the quaraine rotaxane shuttle and its shuttle behavior. (B) Chemical structure of the C_60_ fullerene rotaxane shuttle and its shuttle behavior controlled by anions. Photographs are the corresponding fluorescence with different anions under a laser. (C) Chemical structure and schematic illustration of *trans*‐*cis* isomerization of the perylenebisimides linked alkene molecular motor. Photographs are the corresponding fluorescence of *trans* and *cis* state. *Copyright © 2010, American Chemical Society*. (D) Chemical structures of the phosphorescent rotaxane shuttle and its shuttle behavior controlled by acid and base.

For the non‐emitting molecular machine units, wavelength changes can also be achieved by inducing chromophores. Smith et al.^[105]^ reported their creation of a molecular shuttle, composed of a bis‐anthracene‐containing tetralactam macrocycle and an axle‐containing squaraine dye (Figure 8a). Initially, the macrocycle had a tendency to remain at the central squaraine station, leading to a green solution as a result of charge transfer between the anthracene and squaraine. However, when chloride anions were introduced, the macrocycle was transported away from the central squaraine station, resulting in a disrupted charge transfer interaction and a color shift to blue, which could be completely reversed by removing the chloride anions. The alteration in color served as conclusive evidence for the relocation of the molecular shuttle.

Employing a comparable molecular shuttle approach, Beer et al.^[106]^ synthesized a [3]rotaxane molecular shuttle that changed the fluorescence (Figure 8b). This molecular shuttle consisted of a central C_60_ fullerene bis‐triazolium axle with four‐station bis‐naphthalene diimides and two ferrocenyl‐functionalized isophthalamide macrocycles by using an anion template synthetic methodology.^[107]^ In the presence of chloride anions, the ferrocenyl macrocycles resided at the center of the axle component, causing emissions by the naphthalene diimides fluorophore without charge transfer interactions with the ferrocene units. Conversely, when hexafluorophosphate anions were utilized instead of chloride anions, the ferrocenyl macrocycles relocated to the naphthalene diimides stations, resulting in a decrease in fluorescence emissions. Based on the alkene molecular motor, Feringa et al.^[108]^ devised a molecular machine that could convert monomer to excimer through photochemical means (Figure 8c). The machine consisted of two perylenebisimides, connected through alkyl spacers to prohibit through‐bond electronic communication. In the *trans* state, the perylenebisimide groups exhibited little intermolecular interaction, and the spectral features were consistent with those of perylenebisimide monomers. Upon irradiation at 312 nm, the machine was photochemically switched to the *cis* state, causing the two perylenebisimides aligned in a co‐facial configuration. This led to characteristic modifications in the UV‐Vis spectra indicative of excimer formation, which could be reversed by exposure to 365 nm irradiation.

Phosphorescence, a special form of fluorescence, can also serve as evidence that molecular machines are in motion. Tian et al.^[109]^ developed a [2]rotaxane with a Pt(II) porphyrin‐containing crown ether macrocycle threaded onto two different recognition sites (ammonium salt and 4, 4′‐bipyridinium), with the anthracene moiety acting as one of the terminal stoppers (Figure 8d). In its protonated state, the macrocycle was located near the anthracene unit at the ammonium salt site, and the porphyrin unit exhibited strong room temperature phosphorescence emission due to the distance‐dependent charge transfer interaction between the anthracene and porphyrin. However, the deprotonation of the ammonium salt unit could displace the macrocycle to the 4, 4′‐bipyridinium station, causing a significant reduction in room temperature phosphorescence emissions.

### 5.2 Circularly polarized luminescence

In recent decades, the use of chemical methods to emit left‐ and right‐handed circularly polarized light with different intensities through the non‐racemic chiral luminescence system, also known as CPL, has become a frontier of worldwide research.[^110^, ^111^] Recently, there have been some designed and prepared molecular machines related to CPL as well. The dissymmetry factor (*g*_lum_) is a key parameter that can characterize CPL and is also becoming an important way of observing molecular machines.

**FIGURE 9:**
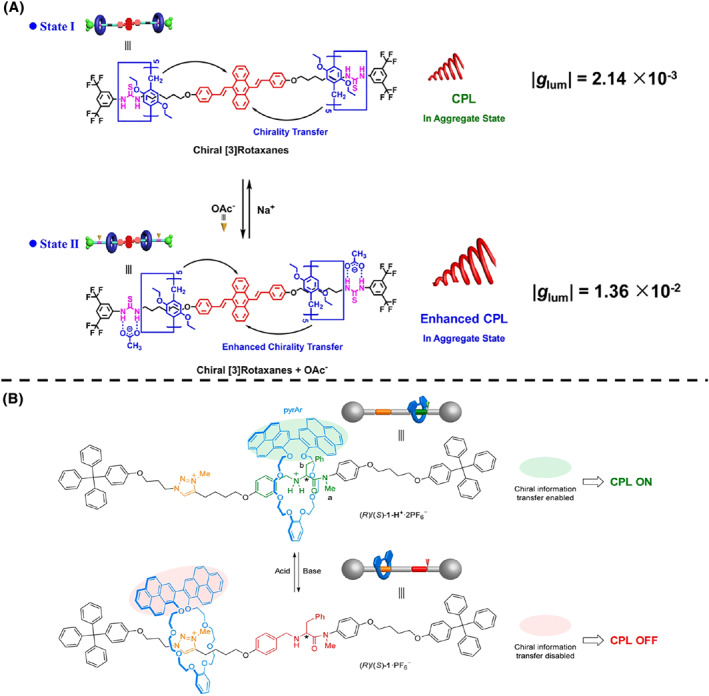
(A) Chemical structure and schematic illustration of the CPL rotaxane shuttle and its shuttle behavior controlled by anions. *Copyright © 2021 Wiley‐VCH GmbH.* (B) Chemical structure and schematic illustration of the CPL rotaxane shuttle and its shuttle behavior controlled by acid and base. *Copyright © 2019, American Chemical Society*.

Yang et al.^[112]^ investigated a chiral [3]rotaxane composed of a fluorescent 9, 10‐distyrylanthracene unit, two thiourea units, and two pillar[5]arene macrocycles (Figure 9a). In the initial state, the pillar[5]arene macrocycles were expected to be located at the thiourea units due to stronger hydrogen‐bonding interactions between the thiourea and ethoxy group of the pillar[5]arene macrocycle, and the |*g*_lum_| value was 2.14 × 10^−3^. When acetate anions were introduced, the pillar[5]arene macrocycles moved away from the neutral alkyl chain and closed to the chromophore, due to thiourea's excellent hydrogen‐bonding donor capability for the acetate anion. This structural change resulted in an enhanced CPL response, and the |*g*_lum_| value increased to 1.36 × 10^−2^.

Blanco et al.^[113]^ demonstrated a [2]rotaxane comprising a crown ether‐derived macrocycle with a 2, 2′‐bipyrene unit and a thread containing an ammonium salt unit derived from *L*/*D*‐phenylalanine and *N*‐methyltriazolium as another binding site for the macrocycle (Figure 9b). In its initial state, the ammonium unit was the preferred binding site, resulting in a CPL emission with a |*g*_lum_| value of ∼0.5 × 10^−3^ due to chirality transfer from the chiral stereogenic unit of *L*/*D*‐phenylalanine. However, upon the addition of a base, *N*‐methyltriazolium exhibited a stronger binding affinity, causing the macrocycle to move away from *L*/*D*‐phenylalanine. This resulted in reduced CPL emission with a weaker influence from the chiral amino acid residue, as the distance between the macrocycle and the chiral center on the thread increased.

## 6 CATALYTIC PERFORMANCE

Owing to their dynamic properties and special topological structures, molecular machines are ideal scaffolds to construct catalysts that can be regulated by an external stimulus in various chemical processes.[^114^, ^115^, ^116^, ^117^] Benefiting from developments in both catalysis and supramolecular chemistry, sophisticated catalytic molecular machines that can perform advanced tasks, such as turning catalytic reactions “on” and “off”, have been explored.^[118]^

**FIGURE 10:**
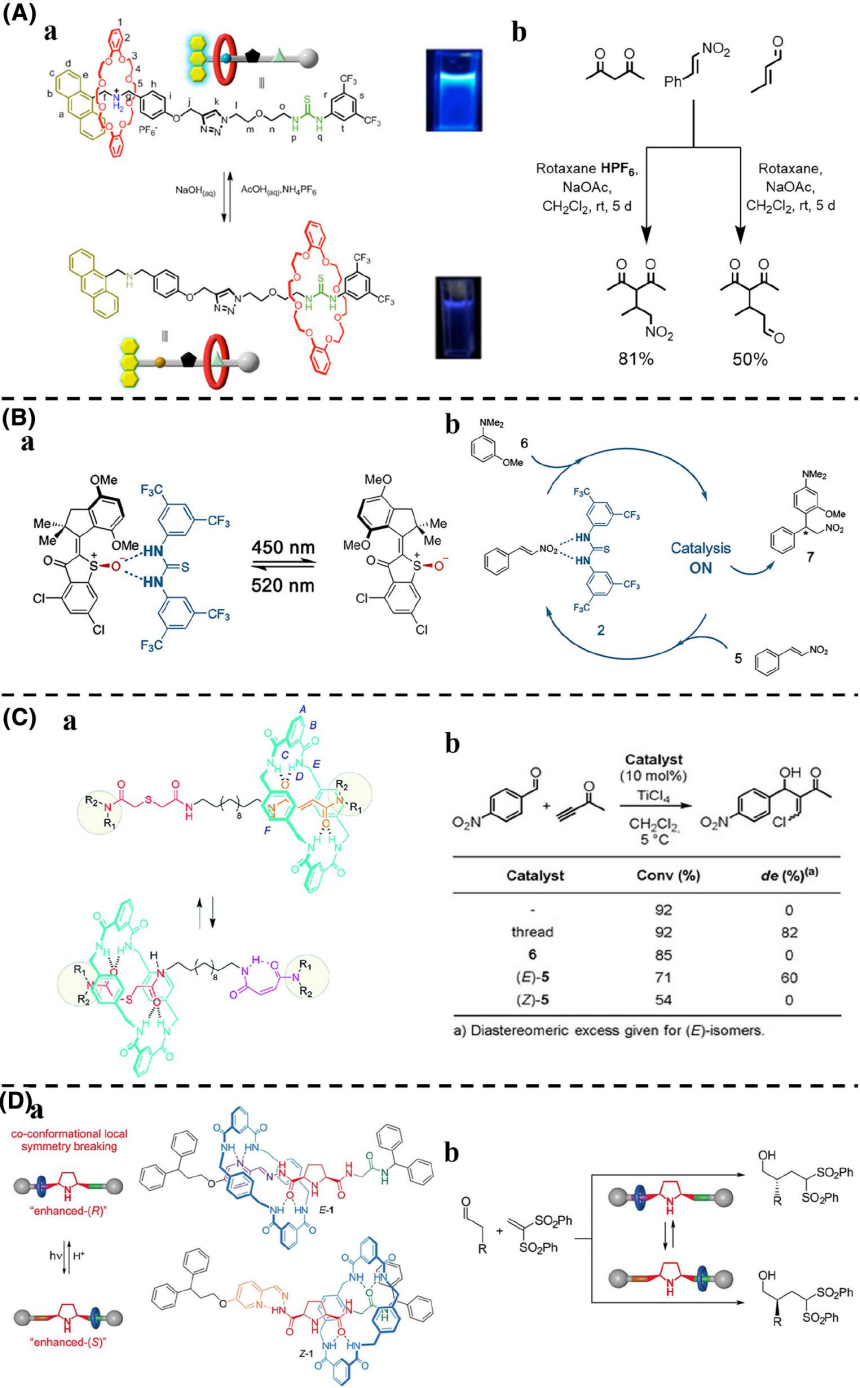
(A) (a) Chemical structure and schematic illustration of the rotaxane shuttle with two catalytic sites (the amine and thiourea). Photographs are the corresponding fluorescence of the rotaxane shuttle in different states. (b) Catalytic performance of the rotaxane shuttle in different states. *Copyright © 2016, American Chemical Society*. (B) (a) Chemical structure and schematic representation of the hemithioindigo‐based molecular motor and its tunable binding with Schreiner's thiourea catalyst. (b) Catalytic reaction using Schreiner's thiourea catalyst. *Copyright © 2020, American Chemical Society*. (C) (a) Chemical structure of the light‐driven rotaxane shuttle. (b) Catalytic reaction using the rotaxane shuttle catalyst in different states. *Copyright © 2017, Royal Society of Chemistry*. (D) (a) Chemical structure and schematic illustration of the rotaxane shuttle controlled by light and acid. (b) Stereoselective catalytic reaction using the rotaxane shuttle catalyst in different states. *Copyright © 2019 Wiley‐VCH Verlag GmbH & Co. KGaA, Weinheim.*

Leung et al.^[119]^ synthesized a [2]rotaxane shuttle with two catalytic sites, an amine and a thiourea, which could be switched by acid and base. The crown ether macrocycle masked either the thiourea or the amine under different pH conditions, resulting in acid‐base switchable catalytic properties (Figure 10Aa). At low pH, the thiourea unit catalyzed the formation of the nitro compound with 81% yield, while the amine was shielded by the macrocycle (Figure 10Ab). At high pH, the amine unit was exposed and the thiourea unit was masked, resulting in the formation of aldehyde compound with a yield of 50%. Therefore, the major product determined the state of the [2]rotaxane shuttle.

In addition to possessing catalytic properties, molecular machines are able to selectively recognize catalysts to switch on/off catalytic performance. Dube et al.^[120]^ presented a modular supramolecular approach to translate the photoinduced geometry changes of a hemithioindigo‐based molecular motor into the catalytic efficiency of a chemical reaction (Figure 10Ba). In the *trans* isomer, weak hydrogen bonding interactions were observed between the molecular motor and Schreiner's thiourea catalyst,^[121]^ while in the *cis* isomer, a more than 10‐fold modulation of the binding affinity was shown. Consequently, when the *trans* isomer of the molecular motor and Schreiner's thiourea catalyst was added to a Michael addition reaction between nitrostyrene and 3‐methoxy‐dimethylaniline, the nitro group of the nitrostyrene and the catalyst were bound via hydrogen bonding, increasing the electrophilicity of the adjacent double bond and facilitating the reaction within hours at a 20% catalyst loading (Figure 10Bb). Upon photoisomerization of the molecular motor, the *cis* isomer was favored to bind to Schreiner's thiourea catalyst, significantly slowing the reaction, and resulting in only partial conversion within the same time frame.

Molecular machines can exhibit a tunable stereoselective catalytic performance by exploiting their ability to transform chirality. In a study conducted by Berna et al.,^[122]^ a photoswitchable rotaxane shuttle consisted of an amide‐based macrocycle, which could be shifted between the fumaramide station and the thiodiglycol amide station of the thread (Figure 10Ca). The (*E*)‐form of the macrocycle displayed a preference for binding to the fumaramide station, while the (*Z*)‐isomer, obtained through light irradiation, favored the thiodiglycol amide unit. When applied to the titanium‐mediated Baylis‐Hillman reaction between aldehydes and alkynes, the macrocycle was initially located around the sulfide and exhibited no reactivity (Figure 10Cb). On the other hand, the rotaxane shuttle was allowed to efficiently switch to the (*E*)‐form and the macrocycle occupied the fumaramide station, thereby enabling the sulfide to act as a nucleophile, resulting in a 60% *de* for the catalytic reaction. However, photoswitching to the (*Z*)‐form led to a complete loss of stereoselectivity, as the sulfide‐station was now blocked.

Leigh et al.^[123]^ showed a catalyst assembled using a clipping method starting from a pseudomeso thread containing two binding sites (pyridyl‐acyl hydrazone and glycine moieties), one on each side of a catalytically active pyrrolidine backbone (Figure 10Da). The entangled polyamide macrocycle was initially positioned over the pyridyl acyl hydrazone. The position of the macrocycle can be switched toward the glycine moiety by light irradiation (*E* to *Z* isomerization of the pyridyl acyl hydrazone). The reverse *Z* to *E* isomerization was also possible by thermal treatment or addition of catalytic acid. This reversible switching of the position of the macrocycle dynamically broke the local symmetry of the thread (pseudomeso form), allowing the implementation of two catalytically active states. This elegant approach has been used to develop an enantiodivergent enamine‐mediated conjugate addition of aldehydes to a vinyl disulphone. Each isomer of the catalyst controls the sense of enantioselectivity in opposite directions, yielding both possible enantioenriched adducts (Δ*ee* up to 60%), depending on the position of the macrocycle along the thread (Figure 10Db). This strategy effectively enhances the enantiocontrol of a catalyzed process by a stimuli‐induced breaking of the local symmetry of the catalyst, shifting the sense of enantioinduction by changing the environment around the catalytic site, and providing a promising method to observe molecular machines by measuring the chiral signals of the products.

## 7 ELECTRICAL SIGNAL

As the electronics industry is integrating more and more new molecules to utilize them in logic circuits and memories to achieve ultra‐high efficiency and device density, many organic structures emerged as promising candidates either in conjunction with or as an alternative to conventional semiconducting materials such as but not limited to silicon.[^124^, ^125^] Due to the different stimulus responses of molecular machines, they could be excellent molecular electronics^[126]^ and memory materials.^[127]^

**FIGURE 11:**
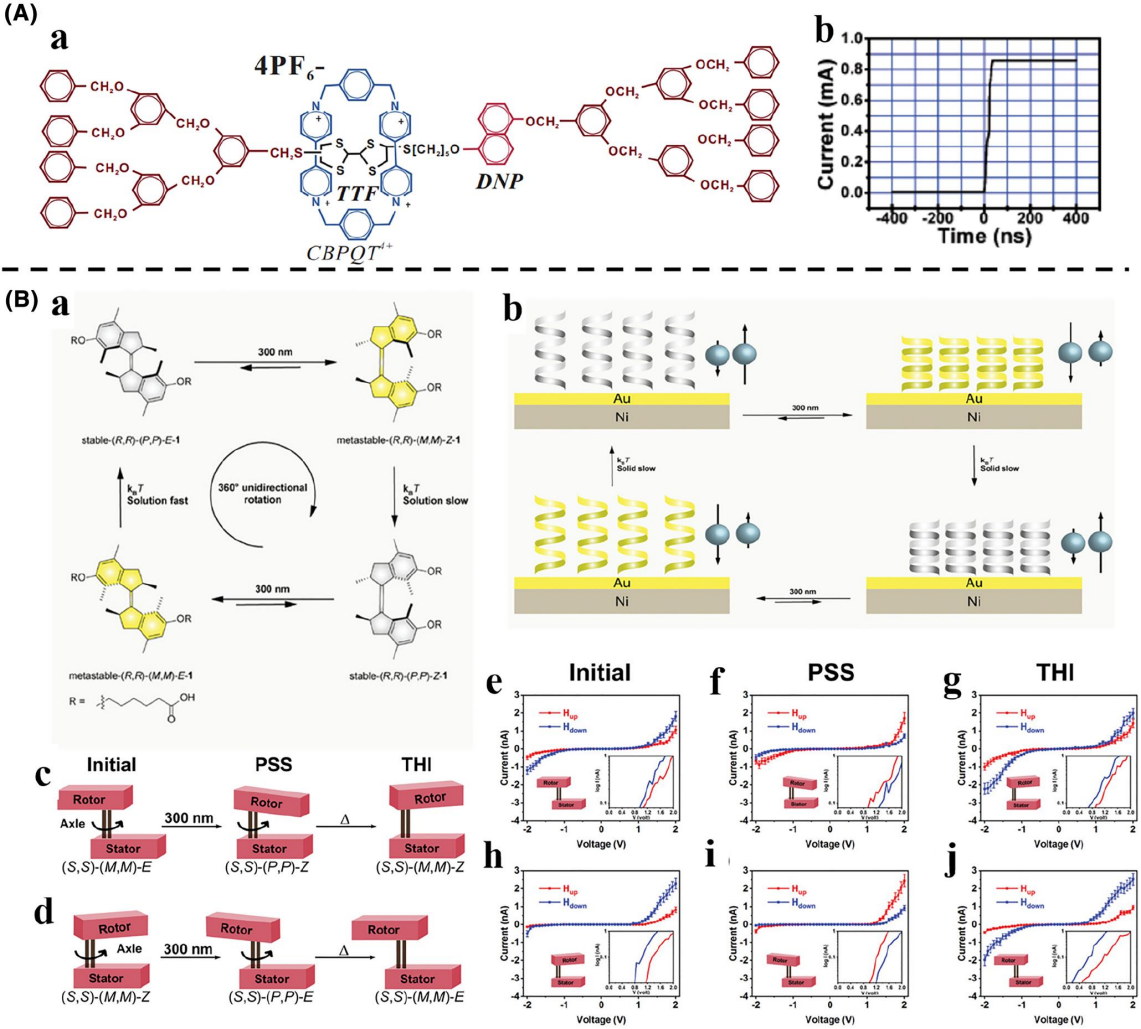
(A) (a) Chemical structure of the conductance‐tunable rotaxane shuttle. (b) Current of the rotaxane shuttle when the conductance switching. *Copyright © 2005, American Chemical Society*. (B) (a) The four‐step rotation of the alkene molecular motor. (b) Schematic depiction of the four‐stage spin polarisation switching in electron tunneling through the molecular motor thin film on nickel/gold (Ni/Au) substrate, which corresponds to the unidirectional rotation through four helical states. (c) Rotary motion around the double‐bond axis of the molecular motor from (*S*, *S*)‐(*M*, *M*)‐*E* to (*S*, *S*)‐(*P*, *P*)‐*Z* and then (*S*, *S*)‐(*M*, *M*)‐*Z*. (d) Rotary motion around the double‐bond axis of the molecular motor from (*S*, *S*)‐(*M*, *M*)‐*Z* to (*S*, *S*)‐(*P*, *P*)‐*E* and then (*S*, *S*)‐(*M*, *M*)‐*E*. (e) Averaged *I*–*V* curves of initial (*S*, *S*)‐(*M*, *M*)‐*E*. (f) Averaged *I*–*V* curves of irradiated (*S*, *S*)‐(*P*, *P*)‐*Z*. (g) Averaged *I*–*V* curves of reversed (*S*, *S*)‐(*M*, *M*)‐*Z*. (h) Averaged *I*–*V* curves of initial (*S*, *S*)‐(*M*, *M*)‐*Z*. (f) Averaged *I*–*V* curves of irradiated (*S*, *S*)‐(*P*, *P*)‐*E.* (g) Averaged *I*–*V* curves of reversed (*S*, *S*)‐(*M*, *M*)‐*E*.

Molecular machines resulted in two stable molecular configurations under external stimuli, known as the “on” and “off” states of the controllable switch with distinct resistances. The motion of molecular machines can be detected by measuring the corresponding currents. Gao et al.^[128]^ designed a thin [2]rotaxane shuttle containing a Langmuir–Blodgett film on a highly oriented pyrolytic graphite surface to achieve stable conductance transitions (Figure 11Aa). The applied voltage changed the oxidation state of the recognition station, driving the macrocycle to move to another station. In this process, the conductivity of the molecule changes, and the corresponding electrical signals become higher or lower (Figure 11Ab). The difference in conductivity between the two states can be used to describe the behavior of the molecular shuttle. This classic example of a voltage‐driven bistable [2]rotaxane shuttle had one CBPQT^4+^ macrocycle and one chain with two electron‐rich recognition sites‐tetrathiafulvalene (TTF) and 1, 5‐dioxynaphthalene (DNP). Normally, the CBPQT^4+^ ring would remain on the tetrathiafulvalene site for a naturally stable state. At this point, the molecule showed a high resistance and low conductance. When the tetrathiafulvalene site was oxidized, the CBPQT^4+^ ring arrived at the 1, 5‐dioxynaphthalene site in a metastable state, showing low resistance and high conductance. When the tetrathiafulvalene site was reduced, and similarly, the 1, 5‐dioxynaphthalene site was oxidized, the CBPQT^4+^ ring backed to the tetrathiafulvalene site and the resistance and conductance changed correspondingly.

In addition to conductance, the spin of the transported electrons can also be controlled in molecular machines to induce current changes. The discovery of spin‐selective electron transport through chiral molecules, i.e., the chiral induced spin selectivity effect, has laid a foundation for the development and construction of spin filters based entirely on organic materials. On this basis, Naaman et al.^[129]^ demonstrated an overcrowded‐alkene molecular motor scaffold acting as a light‐reconfigurable spin filter (Figure 11Ba,b). The molecular motor had four distinct chiral states that can be accessed in a sequential manner in response to light and heat stimuli (Figure 11Bc,d). After being deposited as thin films on gold substrates, molecular motors can still undergo light‐ and heat‐induced isomerization. Four states with distinct PSS and THI can be non‐invasively interconverted in a specific sequence. In the initial (*S*, *S*)‐(*M*, *M*)‐*E*, the averaged current magnitude measured with the magnetic field down was higher than with the magnetic field for all non‐zero voltages. The PSS obtained upon irradiation has opposite chirality, leading to reversal in the observed spin selectivity. Therefore, a higher current was observed. However, (*S*, *S*)‐(*P, P*)‐*Z* was metastable and underwent THI to (*S*, *S*)‐(*M*, *M*)‐*Z* after a few days, as evidenced by the reversal of the spin selectivity to the original preference. For the rest of the cycle, the current showed similar spin selectivity to the curves, reflecting the helicity change at each isomerization step (Figure 11Be–j).

**FIGURE 12:**
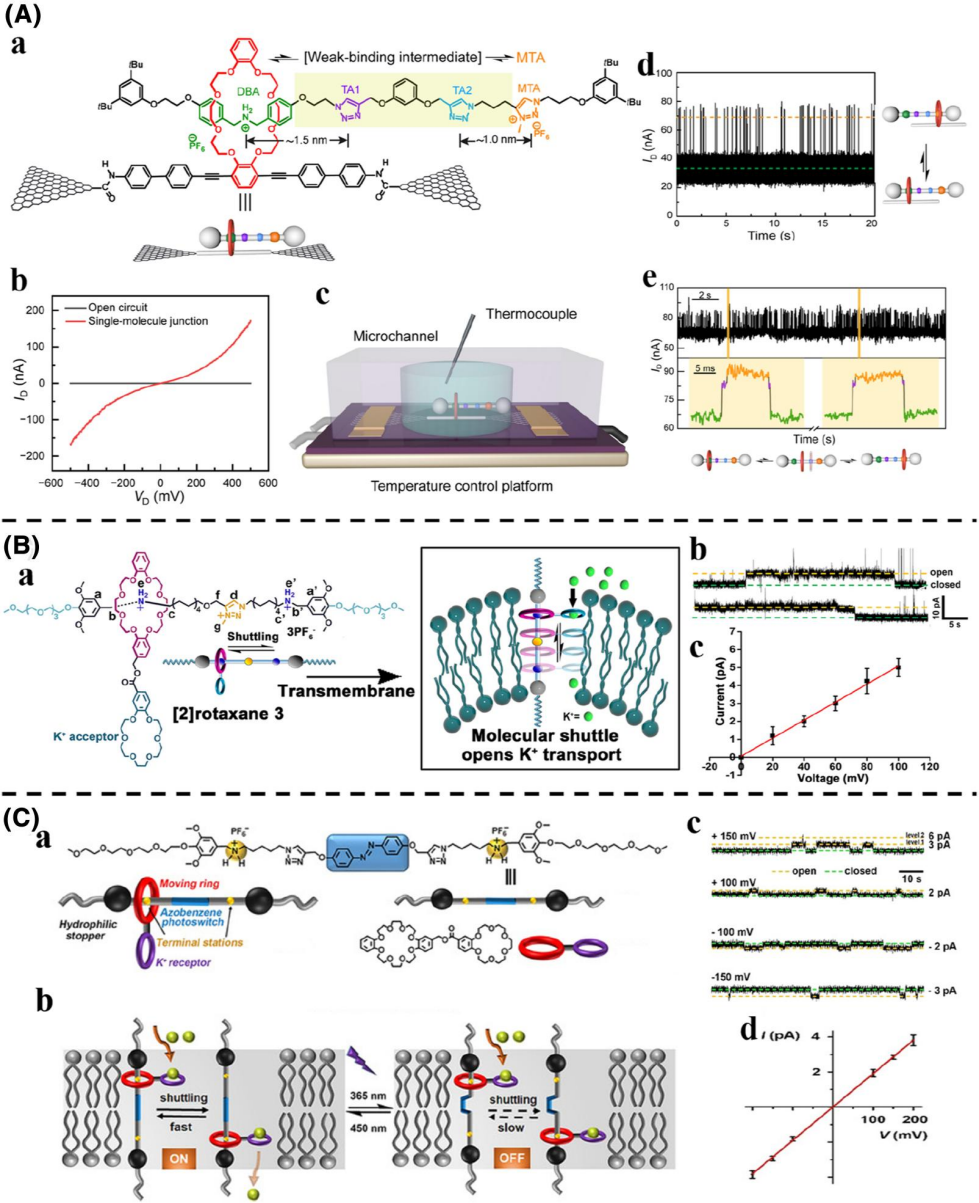
(A) (a) Chemical structure and schematic illustration of the rotaxane shuttle containing diphenylammonium site and triazolium site. (b) *I*‐*V* curves of graphene contacts (black) and the rotaxane shuttle (red) in the solid state. (c) Schematic illustration of GMG‐SMJs technology to monitor the shuttle behavior of the rotaxanes. (d) *I*‐*t* curve of the rotaxane shuttle immersed in CH_3_CN at 298 K for 20 s with a sampling rate of 57.6 kSa/s and schematic illustration of the shuttle behavior of the rotaxanes between diphenylammonium site and triazolium site. (e) Partial *I*‐*t* curve of the rotaxane shuttle immersed in DMSO at 338 K for 20 s with a sampling rate of 57.6 kSa/s, confirming the shuttling process from diphenylammonium site to‐and‐from triazolium via this short‐lived intermediate state was pictured in three steps. *Copyright © 2021 Elsevier Inc.* (B) (a) Chemical structure and schematic illustration of the rotaxane shuttle used in ion transport across membranes. (b) Current recordings of the rotaxane shuttle from a single channel at 100 mV holding potential in a symmetrical 1 M KCl solution. (c) Linear *I*–*V* plots of the rotaxane shuttle with varied holding potentials. *Copyright © 2018, American Chemical Society*. (C) (a) Chemical structure and schematic illustration of the light‐operated rotaxane shuttle used in ion transport across membranes. (b) Proposed mechanism for light‐gated ion transport across lipid bilayers. (c) Representative current recording and (d) linear *I*–*V* plots of the rotaxane shuttle at various holding potentials in a symmetrical 1 m KCl solution. *Copyright © 2021 Wiley‐VCH GmbH.*

Single‐molecule measurement technology, such as single‐molecule force spectroscopy^[130]^ and optical tweezers,^[131]^ can be used to study the dynamic behavior of molecular machines, which can provide information that is submerged by general experimental systems, such as intermediate state processes. Zhang et al.^[132]^ used graphene‐molecule‐graphene single‐molecule junctions (GMG‐SMJs) technology^[133]^ to integrate a [2]rotaxane into graphene single molecule electrical devices and explore the shuttle behavior of individual rotaxanes (Figure 12Aa). The rotaxane shuttle design was based on a crown ether/diphenylammonium system with two triazoles and a triazolium in the center of the axle. Two terminal amines of the crown ether were connected to the carboxyl group of the graphene electrode by acylation reaction. By monitoring the device in acetonitrile solution at 298 K, real‐time changes in current were obtained, reflecting the movement process of the rotaxane shuttle (Figure 12Ab–d). By analyzing the current signal, two current states were presented. Combining energy calculation and transmission spectrum analysis, it was clear that the low state current was attributed to the state of the crown ether ring at the diphenylammonium site, and the high state current was attributed to the state of the crown ether at the triazolium site. The same device was then prepared in a more polar DMSO solution at higher temperature to monitor the movement of the rotaxane shuttle. Under these conditions, the current signal confirmed the appearance of intermediate states where the crown ether remained at the triazole sites (Figure 12Ae). This was due to the weakening effect of high polarity DMSO on the hydrogen bonding between the crown ether ring and different sites, coupled with the high spatial resolution and high temporal resolution of the graphene‐based single‐molecule measurement technology.

Natural transport proteins can regulate the concentration gradient of ions or molecules inside and outside the membrane by controlling the transmembrane transport of life‐related ions or active molecules on the cell membrane or organelle membrane, thereby enabling the normal functioning of living organisms. Some molecular machines that imitate these natural transport proteins are designed to provide a simple model for studying their transport mechanism and to provide potential drugs for treating related diseases. Qu et al.^[134]^ proposed using the shuttle motion properties of rotaxanes to simulate the structure and function of transport proteins for efficient and selective ion transport across membranes (Figure 12Ba). An amphiphilic [2]rotaxane with three binding sites, two ammonium sites and one triazolium site, was designed. The length of the rotaxane molecule was matched to the phospholipid bilayer membrane, and it was inserted and traversed across the phospholipid bilayer membrane. The macrocycle with ion receptor crown ether shuttled through both sides of the membrane by Brownian motion, achieving passive transport of ions. Two possible states of motion in the phospholipid bilayer membrane have been proposed. One is the U‐shaped insertion state (I) and the other is the transmembrane state (II). A voltage clamp measurement was performed across the planar phospholipid bilayer membrane using KCl as the electrolyte. The addition of [2]rotaxane shuttle produced regular square signals with considerably long‐opening times and ohmic *I*‐*V* profiles, indicating the formation of a stable channel or pore across the bilayer membrane (Figure 12Bb,c). It can be concluded that the ion transport of [2]rotaxane shuttle occurred mainly through the unimolecular transmembrane insertion (II) in the lipid bilayer. Furthermore, they then demonstrated another [2]rotaxane shuttle that can be photostimulated to control the rate of transmembrane transport by the introduction of an azobenzene molecular switch (Figure 12Ca).^[135]^ When azobenzene was in a *trans* conformation, the free shuttle of [2]rotaxane between sites can achieve ion transport across the membrane; when photoisomerization was converted to the *cis* conformation, the curved structure hindered the shuttle motion of the [2]rotaxane, thereby preventing ion transport across the membrane (Figure 12Cb). It was confirmed by the current signal that the channel opening probability of the *trans* isomer was much higher than that of the *cis* isomer, thus realising the transmembrane ion transport of light controllable ON/OFF (Figure 12Cc,d).

## 8 SUMMARY AND OUTLOOK

In conclusion, we have summarized that there are many ways of observing artificial molecular machines, some of which rely on modern instrumentation and advanced observational techniques, while others cleverly exploit the structures and properties of molecules. There is no doubt that these tiny machines are challenging to be studied due to their small size and dynamic nature. Fortunately, recent advances in modern instruments and imaging techniques have provided new insights into their structures and functions. We have discussed various experimental instruments, including OM, STM, AFM, NMR, CD, and CPL, and have shown their application in the field of molecular machines. Besides, we highlight the use of defocused OM, variable temperature NMR, 2D NMR and GMG‐SMJ observational technology, which directly demonstrate the real‐time behavior of artificial molecular machines. Furthermore, even if experimental conditions are limited, scientists can also introduce chromophores or chiral groups in their machines to make them more observable.

Despite significant advances in our understanding of molecular machines, there are still many challenges to be overcome in the future. For example, many molecular machines operate in crowded environments, such as cells,[^136^, ^137^] which makes them difficult to be isolated and observed. In addition, for machines that are more dynamic,[^138^, ^139^, ^140^] conformational changes occur on shorter timescales that are more difficult to capture experimentally. It is hoped that advances in computational modeling,^[141]^ along with the continued development of modern instruments, will likely continue to drive progress in this area. Furthermore, new imaging techniques that combine multiple modalities, such as cryo‐electron microscopy, may provide a more direct view of molecular machines.[^142^, ^143^] Ultimately, these will allow us to better observe molecular machines at the microscale, while also enabling us to design molecular machines with greater functionalities, approaching or even surpassing the level of natural molecular machines.

## CONFLICT OF INTEREST STATEMENT

The authors declare no conflicts of interest.

## Data Availability

The data that support the findings of this study are openly available.
